# Simvastatin is associated with a reduced incidence of dementia and Parkinson's disease

**DOI:** 10.1186/1741-7015-5-20

**Published:** 2007-07-19

**Authors:** Benjamin Wolozin, Stanley W Wang, Nien-Chen Li, Austin Lee, Todd A Lee, Lewis E Kazis

**Affiliations:** 1 Boston University School of Medicine, Boston, MA, USA; 2 Center for the Assessment of Pharmaceutical Practices (CAPP), Boston University School of Public Health, Boston, MA, USA; 3 Center for Health Quality Outcomes and Economic Research (CHQOER) Veterans Administration Medical Center (VAMC), Bedford, MA, USA; 4 College of Management, National Cheng Kung University, Taiwan; 5 College of Science, Sungkyunkwan University, South Korea; 6 Midwest Center for Health Services and Policy Research, Hines Veterans Affairs Medical Center, IL, USA

## Abstract

**Background:**

Statins are a class of medications that reduce cholesterol by inhibiting 3-hydroxy-3-methylglutaryl-coenzyme A reductase. Whether statins can benefit patients with dementia remains unclear because of conflicting results. We hypothesized that some of the confusion in the literature might arise from differences in efficacy of different statins. We used a large database to compare the action of several different statins to investigate whether some statins might be differentially associated with a reduction in the incidence of dementia and Parkinson's disease.

**Methods:**

We analyzed data from the decision support system of the US Veterans Affairs database, which contains diagnostic, medication and demographic information on 4.5 million subjects. The association of lovastatin, simvastatin and atorvastatin with dementia was examined with Cox proportional hazard models for subjects taking statins compared with subjects taking cardiovascular medications other than statins, after adjusting for covariates associated with dementia or Parkinson's disease.

**Results:**

We observed that simvastatin is associated with a significant reduction in the incidence of dementia in subjects ≥65 years, using any of three models. The first model incorporated adjustment for age, the second model included adjusted for three known risk factors for dementia, hypertension, cardiovascular disease or diabetes, and the third model incorporated adjustment for the Charlson index, which is an index that provides a broad assessment of chronic disease. Data were obtained for over 700000 subjects taking simvastatin and over 50000 subjects taking atorvastatin who were aged >64 years. Using model 3, the hazard ratio for incident dementia for simvastatin and atorvastatin are 0.46 (CI 0.44–0.48, *p *< 0.0001) and 0.91 (CI 0.80–1.02, *p *= 0.11), respectively. Lovastatin was not associated with a reduction in the incidence of dementia. Simvastatin also exhibited a reduced hazard ratio for newly acquired Parkinson's disease (HR 0.51, CI 0.4–0.55, *p *< 0.0001).

**Conclusion:**

Simvastatin is associated with a strong reduction in the incidence of dementia and Parkinson's disease, whereas atorvastatin is associated with a modest reduction in incident dementia and Parkinson's disease, which shows only a trend towards significance.

## Background

Dementia is one of the major public health threats that individuals face as they age. Epidemiological studies suggest that cardiovascular disease, hypercholesterolemia, hypertension and diabetes are important risk factors for the development of dementia [1-4]. Some studies suggest that medications used to treat these risk factors are also associated with a reduced incidence of dementia [5-10]. Statins are a class of lipid-lowering agents that act by inhibiting the second enzyme in the cholesterol synthetic cascade, 3-hydroxy-3-methylglutaryl-coenzyme A (HMG-CoA) reductase. Many studies have investigated whether statin treatment might be of benefit for subjects with dementia or at risk for dementia, but the conclusion remains ambiguous because of conflicting results [9-15].

Initial studies by our group and that of Jick *et al *suggested that statins might be beneficial as a therapy for dementia [9,10]. Subsequent studies using a cross-sectional analytic method consistently observed a reduced prevalence and incidence of dementia among statin users (reviewed by Sjogren *et al*[16]). However, the cross-sectional nature of these studies raises concerns based on the potential for unanticipated biases including confounding by indication. These concerns have prompted investigators to pursue other strategies to test whether statins might protect against dementia.

Two-wave epidemiological studies examined the effects of statins on incident Alzheimer's disease (AD), but failed to show a statistical benefit associated with statin use [11,17]. The number of subjects on statins who developed incident AD in these two-wave studies was only in the single digits, however, which raises concerns about whether they had sufficient power to detect any putative statin effects.

Prospective incidence trials have also been examined. Cognitive arms were added onto ongoing cardiology studies of which the original goal was to test the efficacy of statins in preventing cardiovascular incidents [18,19]. These studies also failed to show a reduction in incident dementia associated with statin use, but concerns can be raised about the use of an add-on design and whether the studies were sufficiently powered to detect small effects of statins on incident dementia.

Two studies were also performed to investigate whether statins might delay the progression of cognitive decline in subjects with mild to moderate AD [12,14]. Both of these studies were quite promising because they showed reduced progression of measures of cognitive function, but they were limited by small cohort sizes.

The cumulative review of these various studies leads to a mixed picture, with multiple studies both suggesting and refuting that statins might reduce the incidence or progression of AD.

Powering studies sufficiently to detect modulation of incident dementia by medication presents a significant challenge for investigators in the field because only a small fraction of subjects in any database are using any particular medication, and only 1–2% of those subjects develop dementia over the course of short-term follow-up. Large population databases provide a useful mechanism to address this problem. For instance, the Decision Support System (DSS) database of the United States Veterans Affairs (VA) medical system is a population database that obtains records from medical centers throughout the USA, which contains diagnostic, pharmaceutical and demographic information on approximately 4.5 million subjects [20]. In this study, we used existing information within this database to obtain prospective data that allowed us to test the hypothesis that use of statins is associated with a reduced incidence of dementia. We found that simvastatin is associated with a significant reduction in incident dementia and atorvastatin is associated with a more modest reduction that is of borderline significance.

## Methods

### Database

The DSS database contains records from FY 2002 to the present, although daily records of prescription utilization are only present from 2003 to the present [20]. We restricted out study to the years 2003–2005 to allow us to track prescription usage for every subject. This strategy also allowed 2002 to be used as a baseline period for our study to ensure that the subjects did not have a prior diagnosis of AD and PD. The database contains records on approximately 4.5 million subjects and approximately 110 million prescriptions annually. The subjects are 94.4% male and 5.6% female [21].

### Exclusion criteria and restrictions

Our analysis was restricted to subjects ≥65 years of age, who did not have a prior diagnosis of AD [International Classification of Diseases, revision 9 (ICD-9) code 331.0], as judged by the absence of a diagnosis of AD during the baseline period of analysis from 2002–3. For the studies of Parkinson's disease (PD), a prior diagnosis of PD (ICD9 code 332.0) was an exclusion criterion.

Subjects selected for further analysis were also those who had 7 months of continual use of statins after initiation of pharmacotherapy (a time period that we refer to as the treatment window), without diagnosis of AD during this period. Continual use was defined by the presence of repeat prescriptions refills during the 7-month time period, with gaps in prescription refills that were of no more than 6 weeks in duration. A floating starting point was used for a subject to establish the exclusion criteria for a subject and we determined the time to a censoring event for a subject prospectively. Subjects were analyzed for continual use of medication only during the first 7 months of analysis. A similar approach was used for establishing newly acquired Parkinson's disease, using ICD9 code 332.0.

### Strategy for analysis

We selected for the ICD9 code 331.0, defined as "senile dementia of the Alzheimer type". The diagnosis of AD (ICD 331.0) in the DSS database is not rigorously controlled and in many cases may not fulfill all of the National Institute of Neurological Disorders and Stroke/Alzheimer's and Related Disorders Association (NINDS-ARDA) criteria required for diagnosis of AD, because the DSS database is a population-derived database. To acknowledge this diagnostic ambiguity, we have used the term dementia, rather than AD throughout this report. Prior studies of the VA indicate that the accuracy of the diagnosis of AD according to the ICD9 331.0 code is 70–95%, depending on the medical center (A. McKee, personal communication). The ICD9 code 332.0 was used for the diagnosis of PD.

Medications were selected using codes derived from the national veterans affairs formulary. Covariates previously linked to AD were used for the analyses. These covariates used were age, hypertension (ICD9 401–405 and 459.3), cardiovascular disease (ICD9 277.7, 429.2, 410–414, 428, 430–438, 440 and 794.3), diabetes (ICD9 250) and obesity (ICD9 278). The covariates used for analysis of PD were similar to those for dementia, except that we also included use of neuroleptic medications as a covariate (aripiprazole, clozapine, olanzapine, resperidone, haloperidol, chlorpromazine, fluphenazine, perphenazine, thioridazine, thiothixene and trifluoperazine). The covariates were measured during the entire analysis period.

### Comparators/models

We used two different approaches to identify reference groups as comparators for the analyses of subjects taking statins. The age distribution of each comparator group was matched to that of the statin group by identifying records of subjects ≥65 years of age, dividing the subjects into decades (65–74, 75–84, 85–94, ≥95) and determining the proportion of subjects in each age group. The comparator groups were categorized by age in a similar manner, and the same proportion of subjects from each age group was identified. We determined the mean age of subjects in each of the comparator groups, as well as the Charlson comorbidity index and number of hospital admissions in the groups [22].

#### Cardiovascular comparator

We used a cardiovascular comparator as the primary comparator because many statin users have comorbid cardiovascular disease. Statin use is frequent among subjects with cardiovascular disease, and cardiovascular disease is considered to be one of the major risk factors for dementia. In total, 400000 records were randomly selected from subjects taking any cardiovascular medication, excluding those taking statins, using the proportional division among age groups described above. We chose to use cardiovascular medication as a criteria rather than any cardiovascular diagnosis to select for subjects who were sufficiently responsive to the health care system to take medications, which is analogous to those taking statins.

Warfarin was used as a secondary comparator to allow comparison to a specific medication, because use of one identified medication facilitates assessment of potential modifying factors. Warfarin was chosen because it has not been reported in the literature to modify the course of dementia.

### Statistical analyses

SAS software (version 9.01; SAS Institute, Cary, NC, USA) was used for all statistical calculations. Kaplan-Meier survival curves were plotted to show the rate of events. Cox proportional hazards models were used to estimate the association between exposure to statins and risk of dementia. The covariates described in the analysis section were included in these models. Point estimates and 95% confidence intervals (CI) are reported for the adjusted hazard ratios (HRs). We analyzed several different models that incorporate increasing numbers of interaction terms. The results of each model are described in Tables 2 and 4.

**Table 1:** Characterization of subjects taking statins or in comparator groups.

Drug Name	No. of cases	Age		Average Charlson Index		Average no. of hospital stays		Cases (%)
			
		Mean	SD	Mean	SD	Mean	SD	
CV	394 739	74.6	5.7	2.0	2.0	0.5	1.5	30.6
Warfarin	53 369	75.9	5.7	2.6	2.3	0.8	1.9	4.1
Atorvastatin	53 869	73.5	5.3	2.3	2.2	0.6	1.6	4.2
Fluvastatin	5 136	74.2	5.6	2.0	2.1	0.4	1.2	0.4
Lovastatin	54 052	74.8	5.6	2.1	2.1	0.4	1.4	4.2
Pravastatin	1 778	73.5	5.3	2.4	2.4	0.5	1.4	0.1
Simvastatin	727 128	74.5	5.6	2.0	2.0	0.4	1.3	56.4
Total	1 290 071	74.6	5.6	2.1	2.0	0.4	1.4	100.0

Column 1, medication group; column 2, the number of subjects ≥65 years who exhibited continuous use of statins for at least 7 months; columns 3 and 4, mean age and standard deviation (SD); columns 5 and 6, Charlson Index and SD; columns 7 and 8, hospitalization rate and SD; column 9, percentage of cases in each group.

**Table 2:** Models used for analysis of the effects of statins on incidence of dementia using the Cox proportional survival method.

	Model 1				Model 2				Model 3			
	
Variable	HR	p value	95% Confidence limits for HR		HR	p value	95% Confidence limits for HR		HR	p value	95% Confidence limits for HR	
Atorvastatin												
Versus CV	0.80	0.0005	0.71	0.91	0.90	0.09	0.79	1.02	0.91	0.11	0.80	1.02
Age					1.11	<0.0001	1.10	1.12	1.11	<0.0001	1.10	1.11
Hypertension					1.10	0.0590	1.00	1.20				
CVD					1.13	0.0005	1.05	1.20				
Diabetes					1.12	0.0009	1.05	1.20				
Charlson Index									1.78	<0.0001	1.66	1.91
Lovastatin												
Versus CV	0.98	0.70	0.89	1.08	0.95	0.32	0.86	1.05	0.95	0.34	0.86	1.05
Age					1.11	<0.0001	1.10	1.12	1.11	<0.0001	1.10	1.11
Hypertension					1.09	0.07	0.99	1.19				
CVD					1.12	0.0005	1.05	1.20				
Diabetes					1.12	0.0009	1.05	1.20				
Charlson Index									1.74	<0.0001	1.62	1.86
Simvastatin												
Versus CV	0.45	<0.0001	0.43	0.47	0.45	<0.0001	0.43	0.48	0.46	<0.0001	0.44	0.48
Age					1.11	<0.0001	1.10	1.11	1.11	<0.0001	1.10	1.11
Hypertension					1.07	0.07	1.00	1.15				
CVD					1.09	0.0012	1.04	1.15				
Diabetes					1.08	0.0034	1.03	1.14				
Charlson Index									1.72	<0.0001	1.63	1.81

HR, hazard ratio, CVD, cardiovacular disease, CV, cardiovascular.

Model 1 included an adjustment for the interaction between dementia and age; model 2 included adjustments for the interaction between dementia and three disorders associated with increased risk of dementia.; model 3 included adjustments for the interaction between dementia and the Charlson Index, which is an index that provides a broad assessment for the amount of chronic disease. For the purpose of analysis of interactions with the Charlson Index, subjects with a Charlson Index of 0 or 1 were compared with those with a Charlson Index of ≥2.

**Table 3:** Statistical parameters characterizing the results obtained from Kaplan-Meier survival curves. Characterization of Dementia Incidence in the Statin Groups and CV Comparator

		Dementia cases		Time to incidence for dementia cases (months)		Time to end of study for non-dementia cases (months)		
					
Drug	Sample size (n)	n	%	Mean	SD	Mean	SD	Model 3: HR for incidence of dementia vs CV (95% CI)
CV	394 739	3359	0.85	19.8	8.0	30.3	8.6	
Atorvastatin	53 869	275	0.51	19.5	7.7	23.9	11.6	0.91 (0.80–1.02)
Lovastatin	54 052	439	0.81	19.1	7.9	29.7	8.1	0.95 (0.86–1.05)
Simvastatin	727 128	2647	0.36	19.3	7.8	29.1	8.0	0.46 (0.44–0.48)

CV, cardiovacular.

Column 1, medication group; column 2, sample size; column 3, number of dementia cases recorded during the course of the analytic period; column 4, percentage of dementia cases (calculated by dividing the value in column 3 by that in column 2); columns 5 and 6, number of months between start of the analytic period and incidence of dementia, and standard deviation (SD); columns 7 and 8, number of months between start of the analytic period and point at which subjects not receiving a diagnosis of dementia were censored due to the end of the analytic period, and SD.

Hazard ratio for incidence of dementia based on the results from model 3 (Table 2).

**Table 4:** Models used for analysis of the effects of statins on incidence of Parkinson's disease using the Cox proportional survival method. (A) Atorvastatin; (B) lovastatin; (C) simvastatin. Model 1 included an adjustment for the interaction between PD and age. Model 2 included adjustments for the interaction between PD, three disorders associated with increased risk of PD and neuroleptic use. Model 3 included adjustments for the interaction between dementia, the Charlson Index and neuroleptic use. For the purpose of analysis of interactions with the Charlson Index, subjects with a Charlson Index of 0 or 1 were compared with those with a Charlson Index of ≥2. Cox Survival Models for Incidence of Parkinson's Disease. A. Atorvastatin vs CV Comparator

	Model 1				Model 2				Model 3			
	
Variable	HR	p value	95% Confidence limits for HR		HR	p value	95% Confidence limits for HR		HR	p value	95% Confidence limits for HR	
Atorvastatin	0.90	0.15	0.79	1.04	0.96	0.59	0.84	1.11	0.99	0.90	0.86	1.14
Versus CV					1.05	<0.0001	1.05	1.06	1.05	<0.0001	1.05	1.06
Age					1.00	0.97	0.90	1.12				
Hypertension					1.18	<0.0001	1.09	1.27				
CVD					1.12	0.0059	1.03	1.21				
Diabetes					5.12	<0.0001	4.62	5.68	4.97	<0.0001	4.48	5.51
Charlson Index									1.33	<0.0001	1.23	1.44
Lovastatin												
Versus CV	1.09	0.15	0.97	1.22	1.06	0.33	0.95	1.18	1.09	0.13	0.98	1.22
Age					1.05	<0.0001	1.04	1.06	1.05	<0.0001	1.04	1.06
Hypertension					0.99	0.85	0.81	1.10				
CVD					1.20	<0.0001	1.11	1.29				
Diabetes					1.13	0.0001	1.05	1.23				
Charlson Index					5.16	<0.0001	4.66	5.71	5.02	<0.0001	4.53	5.55
Simvastatin									1.32	<0.0001	1.22	1.43
Versus CV												
Age	0.50	<0.0001	0.47	0.53	0.50	<0.0001	0.47	0.53	0.50	<0.0001	0.49	0.55
Hypertension					1.05	<0.0001	1.05	1.06	1.05	<0.0001	1.05	1.06
CVD					0.97	0.42	0.89	1.05				
Diabetes					1.21	<0.0001	1.14	1.28				
Charlson Index					1.11	0.0012	1.04	1.18				
Atorvastatin					5.26	<0.0001	4.85	5.70	5.08	<0.0001	4.69	5.50
Versus CV									1.44	<0.0001	1.36	1.53

HR, hazard ratio, CVD, cardiovacular disease, CV, cardiovascular.

Model 1 included an adjustment for the interaction between dementia and age; model 2 included adjustments for the interaction between dementia and three disorders associated with increased risk of dementia.; model 3 included adjustments for the interaction between dementia and the Charlson Index, which is an index that provides a broad assessment for the amount of chronic disease.

## Results

### Characterization of records from the DSS database

Table 1 describes the characteristics of the populations analyzed. The mean ages of the comparators and each of the statins were similar.

We examined the number of hospitalizations and the Charlson Index during the study period, which is an index providing a general assessment of chronic disease [22]. The CV comparator and each of the statins had differences in the Charlson Index that were <4% of the mean standard deviation for the group, and differences in hospitalization rates that were <15% of the mean standard deviation for the group (Table 1). The warfarin group had a hospitalization rate and Charlson Index that were significantly higher than the values for the other groups (Table 1).

We next examined the numbers of subjects taking each medication. Table 1 shows the number of subjects taking statins who were ≥65 years old and had 7 months of continuous use of statin (as defined by continuous prescriptions). Lovastatin, simvastatin and atorvastatin all had large numbers of prescriptions during the 2003–5 period. Fluvastatin and pravastatin showed a modest number of prescriptions, and rosuvastatin was not prescribed to a significant degree in the VA system during the 2003–5. We also analyzed patterns of usage. Use of lovastatin, simvastatin, atorvastatin and pravastatin changed by <50% over the 3 years of analysis. Use of fluvastatin, however, increased approximately 40-fold between 2003 and 2005. The average age of subjects taking statins was 74.6 years, and was similar for the whole group of statin users (Table 1). Based on these data, we did not pursue further studies of pravastatin or fluvastatin. The number of subjects on pravastatin was too few to produce reliable information. Fluvastatin had more subjects but was confounded by a very large increase in use; the large increase in subjects using fluvastatin between the start and end of the analysis meant that the average duration that subjects would be exposed to the medication was much less than for lovastatin, simvastatin or atorvastatin.

### Statins are associated with reduced rate of incident dementia

To determine whether statins were associated with lower rates of incident dementia we examined the cumulative incidence curves for each of the statins using the CV comparator and adjusted for covariates using the hazard rates by the Cox proportional hazard method. We used three different models for the analysis; results are shown in Table 2.

**Figure 1:**
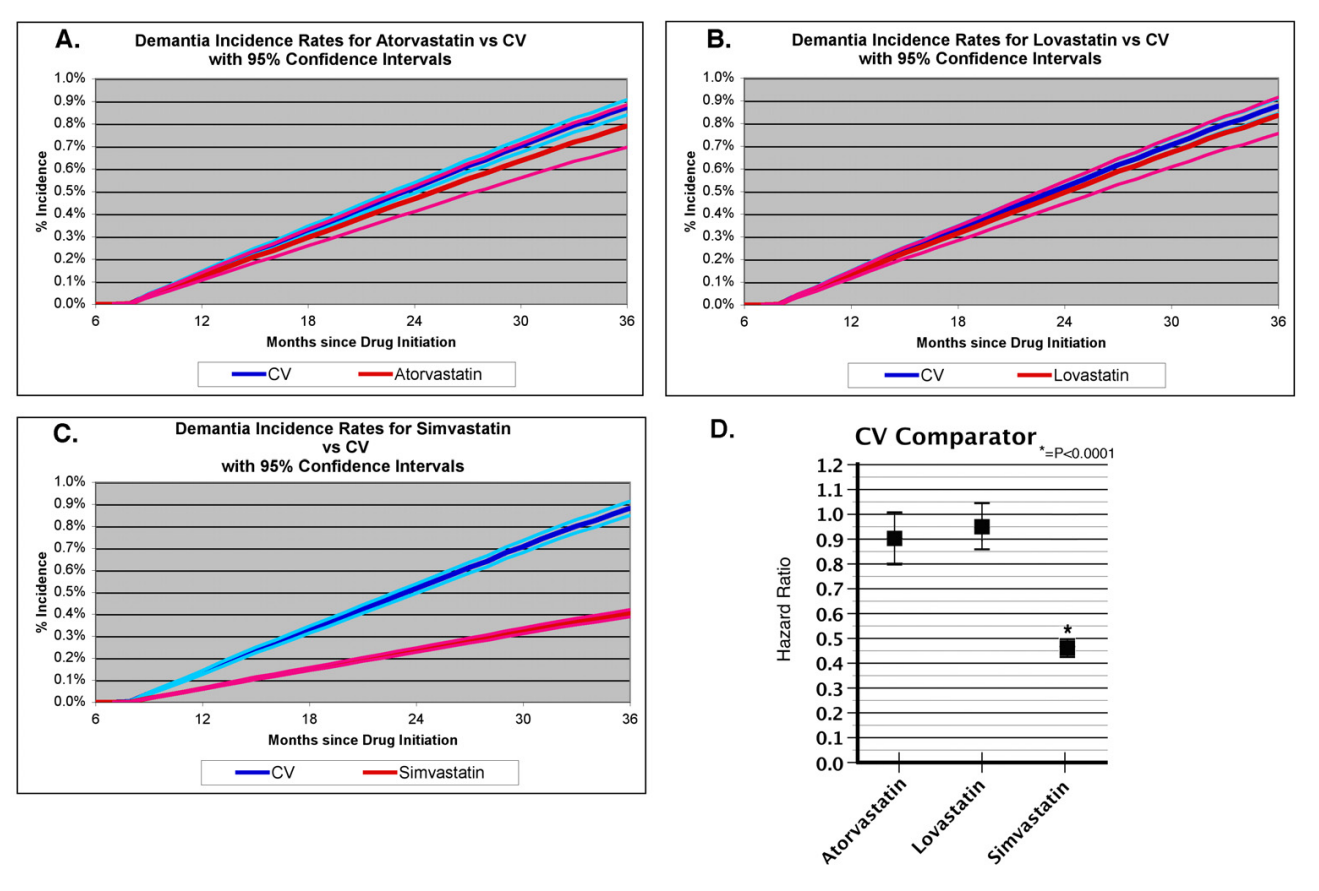
**Cumulative incidence curves for dementia in subjects taking statins**. Cumulative incidence curves are shown for subjects ≥65 years who were taking each statin. The CV comparator was used as the comparison group. Dark blue line, CV comparator; light blue line, confidence intervals for the CV comparator curve; red line, statin group; pink line, confidence intervals for the statin curves. The first 7 months represent the obligate period for drug treatment, during which subjects were treated and any subjects developing dementia were eliminated from the analysis. (**A**) Atorvastatin; (**B**) lovastatin; (**C**) simvastatin; (**D**) summary of hazard ratios for each of the statins using model 3.

Model 1 used an adjustment only for age. Model 2 used adjustments for three major disorders that are known to be important risk factors for AD, cardiovascular disease, hypertension and diabetes. Model 3 used adjustments for the Charlson Index [22]. The adjusted survival curves using Model 3 for the adjustments are shown in Figure 1. Atorvastatin showed a reduction in the HR that was significant in model 1, borderline significant in model 2 (HR = 0.90, CI 0.79–1.02, *p *< 0.1) and not significant in model 3 (Table 2A; fig. 1A, D). Lovastatin did not show a significant reduction in the HRs (Table 2B; fig. 1B, D). Simvastatin showed a strong reduction in the HR for incident dementia that was significant in each of the models, with a HR in Model 3 of 0.46 (CI 0.44–0.48 *p *< 0.0001) (Table 2C; fig. 1C, D). Statistical parameters describing the numbers of cases and censoring are shown in Table 3. The mean time to incident dementia was similar for each of the groups. Cases that reached the end of the study without a diagnosis of dementia were censored. The time to censoring was similar for all groups, except for the atorvastatin group (Table 3). The time to censoring for the atorvastatin group was about 6 months less than that for the other groups, which probably reflects the more recent introduction of atorvastatin to the VA system, leading to a more recent initiation of subjects on atorvastatin than for the other groups (Table 3).

**Figure 2:**
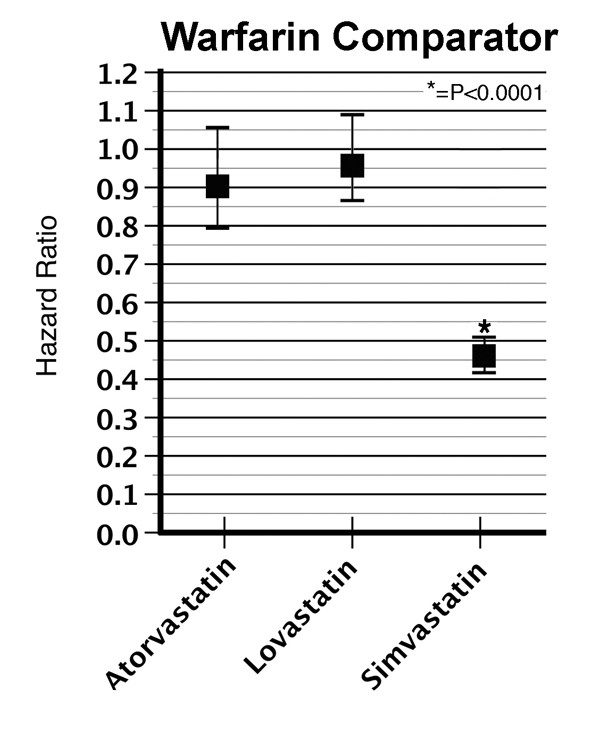
**Cumulative incidence curves using model 3 for incident dementia in subjects taking statins**. The incidence of dementia in subjects ≥65 years were determined for subjects taking each statin with reference to warfarin. The graphical data show the hazard ratios for incident dementia ± 95% confidence interval.

To understand how the type of comparator might influence our results, we also determined the association between statins and odds of incident dementia using a specific medication, warfarin (Figure 2). Simvastatin showed reduced HRs for incident dementia when tested against warfarin. The adjusted HR, using model 3, was 0.46 (CI 0.42–0.51, *p *< 0.0001) against warfarin (Figure 2). Neither atorvastatin nor lovastatin showed a significant reduction in the HR for incident dementia compared with warfarin (Figure 2).

### Statins are also associated with reduced incidence of Parkinson's disease

**Figure 3:**
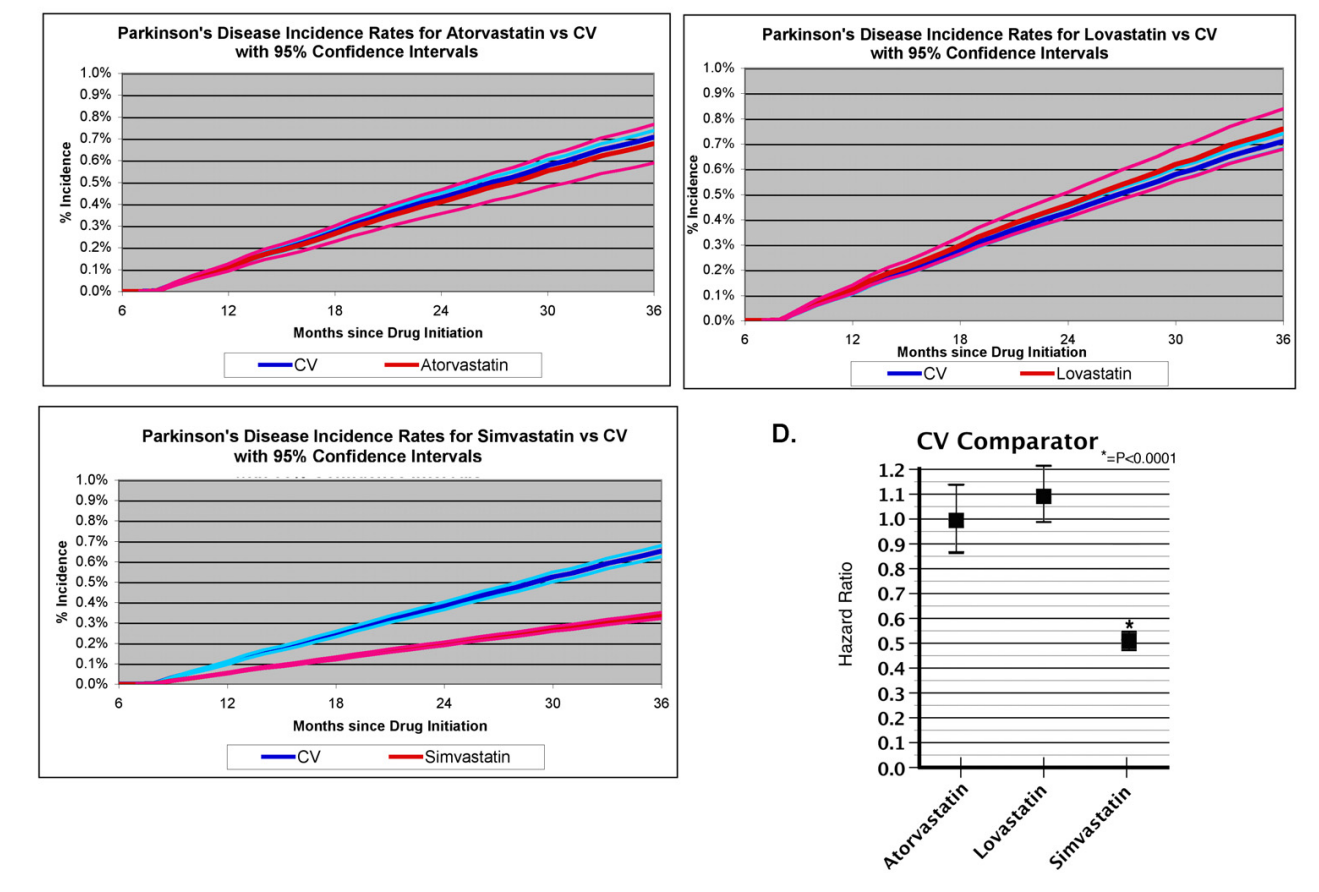
**Cumulative incidence curves using model 3 for incident PD in subjects taking statins (Table 4)**. Cumulative incidence curves are shown for subjects ≥65 years who were taking each statin. The CV comparator was used as the comparison group. Dark blue line, CV comparator; light blue line, confidence intervals for the CV comparator curve; red line, statin group; pink line, confidence intervals for the statin curves. The first 7 months represent the obligate period for drug treatment, during which subjects were treated and any subjects developing dementia were eliminated from the analysis. (**A**) Atorvastatin; (**B**) lovastatin; (**C**) simvastatin; (**D**) summary of hazard ratios for each of the statins using model 3.

The data presented above indicate that statins are associated with a reduced incidence of dementia, but do not provide insight into whether this benefit is selective for dementias or extends to other neurodegenerative diseases. To investigate this question, we examined the effects of statin use on the incidence of PD. The incidence of PD was investigated in subjects taking atorvastatin, lovastatin or simvastatin, and compared with the CV comparator. We adjusted for cardiovascular disease, hypertension, diabetes, and use of neuroleptics. Neither atorvastatin nor lovastatin were associated with significant effects in any of the models (Table 4; Figure 3A, B, D). We observed that simvastatin was also associated with a reduced incidence of PD in all models, with the data for model 3 corresponding to a HR of 0.51 (CI 0.49–0.55, *p *< 0.0001) (Table 4; Figure 3C, D). Statistical parameters describing the numbers of cases and censoring are shown in Table 5. The mean time to incident PD was similar for each of the groups, except for the atorvastatin group, which exhibited a smaller time to censoring (Table 5).

**Table 5:** Statistical parameters characterizing the results obtained from Kaplan-Meier survival curves for PD. Characterization of Parkinson's Disease Incidence in the Statin Groups and CV Comparator

		Dementia cases		Time to incidence for dementia cases (months)		Time to end of study for non-dementia cases (months)		
			
Drug	Sample size (n)	n	%	Mean	SD	Mean	SD	Model 3: HR for incidence of dementia vs CV (95% CI)
CV	392 966	2417	0.62	19.5	8.0	30.3	8.6	
Atorvastatin	53 701	224	0.42	17.9	7.7	24.0	11.5	0.99 (0.86–1.14)
Lovastatin	53 711	350	0.65	18.8	7.9	29.7	8.1	1.09 (0.98–1.22)
Simvastatin	725 820	2116	0.29	19.3	7.9	29.1	8.0	0.51 (0.49–0.55)

Column 1, medication group; column 2, sample size; column 3, number of dementia cases recorded during the course of the analytic period; column 4, percentage of PD cases (calculated by dividing the value in column 3 by that in column 2); columns 5 and 6, number of months between start of the analytic period and incidence of dementia, and standard deviation (SD); columns 7 and 8, number of months between start of the analytic period and point at which subjects not receiving a diagnosis of PD were censored due to the end of the analytic period, and SD.

Hazard ratio for incidence of PD based on the results from model 3 (Table 4).

## Discussion

We previously reported that statins are associated with a reduced prevalence of dementia. Subsequent studies on this subject produced mixed results. We hypothesized that the ambiguity might arise from differences in efficacy among different statins, variation in responses in the population and inadequate sample size to power the studies sufficiently to detect such modifying factors. To address these issues, we used a very large population database to perform prospective studies examining the effects of statins on the incidence of dementia. The prospective format used in the current study avoids effects arising from changes in statin use associated with the diagnosis of dementia, and could account any differences observed between our previous study and this one [9]. We found that simvastatin is associated with a reduced incidence of both dementia and PD.

Studies using population databases have strengths and weaknesses that must be considered in interpreting results. The large numbers of subjects provides enormous power for analyses and the structure of the databases allows for prospective studies of incidence. The strong statistical power associated with the large number of subjects also enables detailed subcategorization of the cohorts, which can provide novel insights. For instance, the ability to subdivide statin users by each statin allowed us to examine the effects of three different statins: atorvastatin, lovastatin and simvastatin. The ability to track subjects over a period of time allowed us to examine incident dementia cases, which represents a significant difference from our previous study on statins, which examined prevalent cases [9]. The focus on incident cases in the current study avoided problems associated with some of the biases that can occur in studies of prevalence, and could explain the differences observed between the two studies [9]. In addition, the covariates provide some adjustment for confounding by indication.

The nature of the prospective study, however, is quite different from that in a randomized clinical trial. Subjects on medications in population databases necessarily have comorbid illnesses, whereas random clinical trials can control the type or degree of comorbid illness or even exclude subjects with comorbid illnesses. Assessing the nature of the sample and the comparators correctly becomes a very important issue in studies examining population databases. We used two comparators, a CV comparator and warfarin. We feel that the CV comparator is the more robust, because the strong link between hypercholesterolemia and cardiovascular disease suggests that the statin group should resemble the CV comparator group. Warfarin was chosen as a comparator to provide a specific medication as a reference, although any particular medication will always have particular concerns relevant to that medication. We chose warfarin because it had not been shown to modify the course of dementia. Warfarin is used to treat many types of vascular disease, including deep vein thrombosis, stroke and atrial fibrillation [23]. Despite use of warfarin to treat vascular disease, referencing simvastatin to warfarin produced results that were substantially similar to those observed when referencing simvastatin to the CV comparator.

Lovastatin users also provided a useful reference, although this group was not designed as a reference at the outset of the study. The incidence of dementia among subjects taking lovastatin was similar to that of the CV comparator, and well above that of the simvastatin group. The case mix of the simvastatin and lovastatin groups might resemble each other more than the case mixes for the other comparators, yet simvastatin showed a greater efficacy for reducing dementia, although it is important to be cognizant that simvastatin is a newer medication than lovastatin, which could lead to a selection bias. The strength of reduction in dementia for simvastatin compared with lovastatin is consistent with many studies showing that simvastatin reduces measures of plasma cholesterol more effectively than lovastatin [24,25]. The presence of a strong and significant difference in HRs for dementia between these two groups (lovastatin and simvastatin) that are congruent for statin use supports the hypothesis that the efficacy of simvastatin arises from the action of simvastatin rather than from a prescription bias.

Analysis of the frequency of hospitalizations and the Charlson Index were used to compare the different groups in this study and used to control for comorbid disease. The CV comparator and each of the statins exhibited similar hospitalization rates and Charlson indices, which suggests that these groups exhibited similar rates of comorbid illnesses. The warfarin group showed elevated Charlson indices and elevated hospitalization rates, which is consistent with a hypothesis that these groups had a higher general rate of morbidity than most of the other groups. The potential differences in comorbid illnesses that might exist among the groups did not exert strong effects on the resulting HRs. When we used models that adjusted for potential interactions between Alzheimer's disease and other chronic illnesses, we did not observe a strong change in the size of the HR describing the relationship between use of simvastatin and incident dementia.

Quantitative indices of subject health, such as laboratory values or imaging studies, are not available in the DSS database, in contrast to databases prepared by academic investigators and in clinical trials [20]. The absence of quantifiable measures means that we were not able to determine the degree of cholesterol reduction associated with each statin, nor were we able to quantify cognition. Although we used the ICD9 diagnostic code for senile dementia of the Alzheimer type, 331.0, misdiagnosis occurs, thus we used the generic term, dementia. Diagnoses of AD in population databases are also not rigorously controlled, and the diagnostic measures often do not meet the NINDS-ARDA criteria for AD. Previous studies indicate that the diagnoses of AD in the VA databases are 70–95% accurate [26]. The presence of ≥70% of cases that probably do have AD suggests that the reduction in incidence of dementia associated with statin use observed in our study also applies to AD, although the exact degree is risk reduction for AD might differ from that observed in our study.

The strength of reduction of incidence of dementia observed with simvastatin is striking. Further studies are required to determine whether this effect represents a biological action of simvastatin or a statistical bias skewing results obtained from the DSS database. If the reduction in incident dementia derives from biological actions of simvastatin, studies in the literature suggest potential biological mechanisms that might contribute to this action. Prior studies indicate that simvastatin is more effective than pravastatin or lovastatin at modifying some measures of lipid metabolism, such as reductions in cholesterol and LDL, and increases in HDL. Simvastatin has a similar efficacy as atorvastatin with respect to reducing measures of lipid metabolism [25,27]. Simvastatin is better that atorvastatin on some measures (e.g., raising HDL), but atorvastatin is better than simvastatin on other measures (e.g., lowering LDL) [25,28-30]. The size of the difference in HR that we observed for simvastatin compared with the other statins appears larger than that observed in studies examining vascular lipid parameters. This raises the possibility that the putative benefit of simvastatin arises from another contributing factor. One factor could be the ability to penetrate the blood-brain barrier. The statins differ in their lipophilicity and ability to cross the blood-brain barrier, with the rank order of permeabilities being lovastatin > simvastatin > atorvastatin [31-33]. Simvastatin is atypical, because of its strong efficacy but intermediate permeability. The combination of lipophilicity and efficacy gives simvastatin a unique pharmacological profile compared with the other statins. These factors might lead simvastatin to be more potent than lovastatin. Atorvastatin has strong anti-inflammatory properties, but the inability of atorvastatin to penetrate the blood-brain barrier might reduce its efficacy in preventing neurodegenerative disease [35]. Further studies are needed to clarify this issue.

The ability to examine multiple disorders is a salient strength of population databases such as the DSS database. In this study, we also examined the efficacy of statins towards PD. The association of statins with reduced incidence dementia and PD raises the possibility that the action of statins against these diseases shares a common mechanism. Many investigators have noted that statins reduce inflammation, osteoporosis, and fractures, as well as diseases directly caused by heart disease [40-42]. Inflammation contributes to the pathophysiology of AD and PD. We recently observed that subjects with severe AD pathology who were taking statins show less inflammation than those not taking statins [43]. It is possible that the putative ability of statins to reduce inflammation contributes to the reduction in incidence of degenerative disease associated with simvastatin.

## Conclusion

Simvastatin is associated with a significant reduction in incident dementia and incident Parkinson's disease. Atorvastatin is associated with a modest reduction in incident dementia that is of borderline significance when age is included as a covariate, but insignificant when other comorbid diseases are included as covariates. Further studies are required to determine whether this effect represents a biological action of simvastatin or an unanticipated statistical bias present in the DSS database.

## Competing interests

BW has a patent for use of statin as therapy for Alzheimer's disease.

## Authors' contributions

BW conceived of the project, analyzed the data and was the primary author of the manuscript. SWW and NCL carried out the computer analyses. AL provided statistical analysis for the study. TAL participated in the design of the study and analysis of the data. LEK participated in the conception of the project, the design of the study, analysis of the data and writing of the manuscript.

## Pre-publication history

The pre-publication history for this paper can be accessed here:


